# Understanding the Nurse Champion Concept and the Training Initiatives for Nurse Champions: Protocol for a Scoping Review

**DOI:** 10.2196/80204

**Published:** 2026-01-14

**Authors:** Wilmer J Santos, Ian D Graham, Amanda Vandyk, Gillian Harvey, Janet E Squires

**Affiliations:** 1 School of Nursing, Faculty of Health Sciences, University of Ottawa, 200 Lees Avenue, Room 313, Ottawa, ON, K1N 6N5, Canada, 1 613-562-5800, ext 5473; 2 Clinical Epidemiology Program, Ottawa Hospital Research Institute, Ottawa, ON, Canada; 3 School of Epidemiology and Public Health, Faculty of Medicine, University of Ottawa, Ottawa, ON, Canada; 4 College of Nursing and Health Sciences, Caring Futures Institute, Flinders University, Adelaide, Australia

**Keywords:** nurse champions, facilitation, health care, implementation, nursing, equity, diversity, inclusion

## Abstract

**Background:**

There is current evidence that a proportion of health care services provided to patients do not align with best evidence. A nurse champion, defined as a nurse who either volunteers or is identified by the management to facilitate or promote the implementation of an innovation (eg, new knowledge or practice), is an important factor for implementation success. The existing literature describes health care champions’ attributes, roles, and behaviors, the processes in which they might enable change, and their effectiveness at facilitating implementation. However, a more detailed exploration of the nurse champion concept is needed. Further, despite the prolific use of nurse champions in health care implementation, there is a gap in the literature pertaining to what nurse champion training initiatives exist, what competencies are important to be a nurse champion, and whether current training initiatives are effective in preparing nurse champions. Finally, the extent to which equity, diversity, and inclusion are considered in the nurse champion literature is unknown.

**Objective:**

This study aims to (1) develop a preliminary conceptual understanding of nurse champions, (2) describe the characteristics of existing champion training initiatives in health care that prepare nurse champions and synthesize the competencies that are covered in these champion training initiatives, (3) synthesize the findings of studies that examined the effectiveness of nurse champion training initiatives in preparing nurses to be effective champions, and (4) evaluate the extent to which equity, diversity, and inclusion are considered in studies that define nurse champions and in studies describing or evaluating nurse champion training initiatives attended by nurse champions.

**Methods:**

This series of linked reviews will follow the Joanna Briggs Institute scoping review methodology. We will systematically search 8 electronic databases using a Peer Review of Electronic Search Strategies. We will also search for gray literature (eg, theses and dissertations). We will upload the records from our database searches into Covidence. Two individuals from the research team will perform title and abstract and full-text screening independently and in duplicate using a piloted inclusion and exclusion criteria. Two individuals will perform data extraction and quality appraisal independently and in duplicate. Conflicts will be resolved with consensus. We will perform various forms of content analysis to address our varying research questions and objectives.

**Results:**

As of January 2026, we have completed more than half of our title and abstract screening. We expect to present the results in a scoping review later in 2026.

**Conclusions:**

The results of this research will provide recommendations for current and future nurse champion training initiatives. Although this review is limited to the nursing discipline, the results may be transferable to understanding champions in other contexts.

## Introduction

### Background

Despite advancement in health research, many authors identify that a significant proportion of care provided to patients is not in line with the available research evidence [1,2]. For instance, Braithwaite et al [1] described the 60-30-10 phenomenon, indicating that about 60% of care aligns with guideline recommendations, that 30% of care is a waste, because it is duplicated, or of low value, and that at least 10% of patients experience iatrogenic harm or adverse events. A more recent systematic review reported that, on average, 30% of care reported in 174 Canadian studies is not concordant with best evidence or guidelines [2].

Knowledge translation is the dynamic and iterative process that is required to synthesize, disseminate, exchange, ethically apply, evaluate, sustain, or coproduce knowledge using theory-informed and tested processes to provide more effective health services and improve the health care system [3,4]. Many authors emphasize the importance of individuals who enable and lead the implementation of care that is in accordance with the best evidence [5-7]. There has been a growing interest and research on the deployment of champions across different contexts in health care [6-12], such as acute care [8], cancer care [9], long-term care [11], and mental health [10]. The champion concept originates from the management business literature in the 1960s and 1970s; champions were initially deployed by companies to disseminate and promote the use of technological innovations [13,14]. Champions are individuals who often volunteer to work above and beyond their occupational role to promote an innovation (eg, new knowledge, practice, policy, and technology) or enact behaviors to mitigate barriers and reinforce facilitators to implementation [6,8,15]. Champions are often health care workers [8,9], but they can also be individuals in management [16-18] or patients [19,20]. Champions might self-identify or emerge organically, or be appointed formally by management [21,22].

There have been ongoing movements to elevate nursing practice so that it is in accordance with the current research evidence, nurses’ experiential knowledge, and patients’ preferences [4,23-25]. The importance of deploying nurse champions is emphasized by researchers across multiple sectors [6,8,26] such as acute care [27,28], primary care [29], and home care [30]. As detailed below, despite the plethora of research on champions, further research is required to understand the concept of nurse champions, how nurse champions are trained, and the equity, diversity, and inclusion (EDI) considerations pertaining to the training and deployment of nurse champions.

### Conceptualization of Nurse Champions

In previous studies, nurse champions have been described as nurses working in a clinical or managerial role who enact facilitation to increase the uptake of an innovation at their place of work [31-34]. Facilitation is defined as the process that activates implementation by assessing and responding to the characteristics of the innovation, the recipient, and the context, thereby emphasizing existing enablers and addressing barriers to implementation [35]. Although there has not been a concept analysis specifically on nurse champions, previous conceptual studies (published in 2006 [36] and 2017 [15]) about health care champions have focused on clarifying terms that refer to individuals who enable implementation, including champions, change agents, coaches, opinion leaders, knowledge brokers, facilitators, and linking agents [15,36]. Authors have reported that there is confusion between the terms used to describe individuals who facilitate implementation [6,15,36]. The same authors have stated that these different terms or concepts are more similar than different, as their main function is often to facilitate successful implementation, but differ based on their intended purpose, how influence is exerted, their domain of influence [36], and their theoretical origins [15].

Authors have described health care champions based on common personal attributes that they possess or the behaviors they enact [6,15]. In a 2017 scoping review n = 195 studies by Cranley et al [15], the authors presented key health care champion attributes and skills (based on 18 papers), which included (1) expert knowledge of the innovation, (2) persuasiveness, (3) mentorship skills, (4) being visionary, (5) enthusiasm, (6) creativity, (7) driven and passionate about their work, and (8) communication skills. In a 2018 integrative review by Miech et al [6], the authors presented a more comprehensive list of characteristics of health care champions that included all the attributes or skills reported by Cranley et al [15] in addition to the following: (1) negotiation skills, (2) advocacy, (3) communication across organizational boundaries, (4) strong educator and presentation skills, (5) having political acumen, (6) leading teams and recruiting new team members, (7) engaging in team planning and goal setting, and (8) collecting data, tracking progress, and providing feedback. In a 2020 comparative case study (n=78 clinicians and health administrators) by Bonawitz et al [37], health care champions have 6 key attributes: (1) they have influence over other people’s opinions and behaviors, (2) they have ownership of the implementation, (3) they are physically present at the point of change, (4) they are persuasive, (5) they have grit, and (6) they enact a participative leadership style.

Authors have formulated conceptual frameworks of champions according to the existing literature [7]. For instance, in a recent conceptual paper published in 2021, Shea [7] presented a conceptual model that describes 7 constructs that pertain to health care champions’ performance and impact in implementation activities. According to this conceptual model, the health care champion’s performance (defined as activities they perform) is influenced by their level of commitment, experience, and self-efficacy. The health care champions’ level of commitment is influenced by their beliefs about the innovation and the organizational support provided to the health care champions. The health care champions’ impact (implementation outcomes [eg, changes in perceived acceptability or appropriateness of the innovation]) is related to the health care champions’ performance mediated by the level of peer engagement with the health care champion during the phases of implementation [7]. Similarly, Morena et al [38] proposed 2 causal pathway models to illustrate how clinical champion attributes and skills, which function under the mechanism of action of social influence, mediate health care providers’ attitudes, health care providers’ self-efficacy, subjective norms, and individuals’ perceived abilities to perform the behaviors, which then at the end leads to increased use of the innovation by health care providers [38].

Although the literature discussed above on champions in health care provides some conceptual understanding that may be informative in understanding the nurse champion concept, a fulsome exploration of the concept is needed. According to Rodgers and Knafl [39,40], conceptual clarity relies on understanding the significance, use, and application of a concept over time and within a particular context. Garnering conceptual clarity on nurse champions is important as there may be varied significance, use, and application of the nurse champion concept within the nursing discipline (eg, management, education, and clinical practice), and there may be changes in how nurse champions have been defined through time. Furthermore, conducting a concept analysis on nurse champions using a systematic process may advance the work of Shea [7], as he reported that his conceptual model was not informed by a systematic review. Finally, as stated by Morena et al [38], the causal pathways they constructed may not be applicable to all clinical champions; hence, an in-depth analysis of nurse champions may reveal nuances pertaining to the attributes of nurse champions.

### Nurse Champions Training Initiatives and Competencies

Despite the prolific use of champions in nursing and in health care, the evidence pertaining to their effectiveness or ability to facilitate successful implementation is variable [6,8]. In a 2018 integrative review (n=199) on champions in health care, Miech et al [6] concluded that deploying champions is a necessary but not a sufficient condition for implementation success. Champions were stated to be necessary because more than 80% of the studies included in their review cited champions as one of the key factors associated with implementation success, but the extent to which champions influenced implementation success, especially with the presence of other implementation strategies, was not clear [6]. Similarly, a 2022 systematic review conducted by members of this team (n=35) [8] on the effectiveness of health care champions in improving innovation use or outcomes within the context of health care implementation found that the presence of champions was associated with the uptake of practices, programs, and technologies at a system or facility level (in 5 of 7, 71.4% studies). However, the deployment of champions was inconsistently correlated with improvements in conceptual (2/4, 50%) and instrumental (8/17, 47.1%) innovation use by health care providers and in improving outcomes for patients (3/6, 50%) [8]. Conceptual innovation use was defined as increased knowledge or improved attitude toward an innovation [41]. Instrumental innovation use was defined as increased use of the innovation [41]. In both reviews [6,8], the authors reported that champions were often operationalized as merely the lack or presence of a champion during implementation activities in health care. Specifically, most of the studies (25/35, 71.4%) included in the 2022 systematic review conducted by members of our research team [8] operationalized champions simply as a dichotomous item reflecting either the presence or absence of someone labeled as a champion. In the same review, the authors [8] reported 5 subscales that were used in 6 studies to evaluate the existence of champions who have particular attributes, or performed specific roles and behaviors. Similarly, Miech et al [6] reported that in more than 90% of the papers included in their integrative review (n=199 articles), champions were reported simply as the presence or absence of a champion. The lack of robust measures pertaining to champions in health care may be attributed to the murky conceptualization of the concept [6,15,36], which further justifies the concept analysis of nurse champions as discussed above. Furthermore, the lack of detailed descriptions of champions or robust measures used in empirical studies evaluating health care champions is problematic because of the variability of roles and behaviors that they can perform [6,9]; these roles and behaviors require different competencies to perform well.

Competency is defined as the ability of an individual to perform a behavior successfully with the necessary attitudes, judgment, knowledge, and skills [42]. A 2025 rapid systematic review of implementation trials [12] (n=15) reported that there was a lack of description of the training provided to champions in health care, according to 12 of the trials (80%) included in their review. Other authors have echoed the concern regarding the lack of understanding of how effective champions in health care are identified and prepared [6,7,38]. Despite the well-synthesized knowledge pertaining to the roles and behaviors of health care champions (which is assumed to be transferable to nurse champions), there is a gap in knowledge pertaining to the training initiatives that prepare nurse champions and the competencies required by nurse champions to perform behaviors that improve innovation use. Furthermore, there is a need to synthesize the evidence pertaining to the effectiveness of nurse champion training initiatives, as this knowledge can inform future implementation studies on what approaches were helpful in producing effective nurse champions.

### EDI Considerations in Training and Deploying Nurse Champions

There was minimal discussion of the EDI considerations pertaining to the role and behaviors of champions in health care in the previous studies discussed above [6-8,12,15,36-38]. In addition, there were minimal or no demographics of the health care champions or the individuals that the champions were intended to influence reported in the studies discussed above [6-8,12,15,36-38]. EDI considerations are important to explore in research pertaining to nurse champions and champions in general because peer or social influence can be more successful at changing behaviors when it is performed by an individual who comes from the same societal group as the people they are trying to influence [43,44]. EDI considerations might also be important to consider when nurse champions or health care champions are deployed to enable the implementation of innovations targeted toward a specific group of people, or when the implementation activity affects equity-deserving populations. For example, in Bonawitz et al’s [37] 2020 comparative case study, obstetrician or gynecologist champions were deployed to promote postpartum contraceptive care, which is an innovation that is specific to women of childbearing age. Morena et al [38] also echo the need to examine champions who work with equity-deserving populations and the EDI considerations in deploying champions.

Leadership styles that have been traditionally associated with gender roles may also influence the deployment of nurse champions. For example, in a 2018 cross-sectional study by Luz et al [21], they hypothesized that men are more likely to be formally appointed by leadership to become nurse champions because men have traditionally been ascribed a leadership style that is more task-oriented, reliant on directing others’ behavior, and is more authoritarian [34,45]. On the other hand, Luz et al [21] hypothesized that women are more likely to be informal nurse champions because women have been traditionally attributed a leadership style that is more collaborative and more reliant on their interpersonal relationships with their peers [46]. Luz et al [21] did not find a relationship between the nurse champions’ gender and whether they were formally or informally appointed; the authors rationalized that the lack of relationship might be related to the small number of men in their sample. Hence, there is a need to examine the extent to which EDI considerations are pertinent to nurse champions’ training, deployment, and behaviors.

### Study Objectives

Our study objectives are to (1) develop a preliminary conceptual understanding of nurse champions, (2) describe the characteristics of existing champion training initiatives in health care that prepare nurse champions and synthesize the competencies that are covered in these champion training initiatives, (3) synthesize the findings of studies that examined the effectiveness of nurse champion training initiatives in preparing nurses to be effective champions, and (4) evaluate the extent to which EDI is considered in studies that define nurse champions and in studies describing or evaluating nurse champion training initiatives attended by nurse champions. Although we acknowledge the conclusions made in previous work that there are a lot of similarities between champions and other concepts (eg, facilitators, change agents, and opinion leaders) [6,15,36], focusing our research on individuals who are explicitly referred to as nurse champions will allow for more nuanced understanding of how this concept has been defined and used in different health care sectors and over time, and what training is being provided to nurse champions.

## Methods

### Study Design

This study is a single scoping review with one prespecified search, screening, eligibility, and data extraction workflow. We will follow the Joanna Briggs Institute (JBI) scoping review approach to conduct this series of link reviews [47], where we will conduct a broad search of the literature, perform varying analyses, and then report the results in 4 papers addressing each of our 4 study objectives. A scoping review is an appropriate methodology because of the broad research objectives. There are five mandatory stages in a scoping review [47,48]: (1) identifying the research question; (2) identifying relevant studies; (3) study selection; (4) data charting; and (5) collating, summarizing, and reporting results. Our review is rooted in a pragmatist paradigm because we are including and gathering knowledge developed using different methodologies and from different sources (ie, published and gray literature) to achieve our research objectives. JBI’s approach in evidence implementation and synthesis is also situated within a pragmatist paradigm [49], which demonstrates alignment between our chosen methodology and paradigm. As detailed in a later section, we will follow specific approaches for identifying relevant articles and perform varying data analysis approaches relevant to each review.

### Identifying the Research Question

There are 11 research questions for the scoping review: (1) What are the definitions used in the literature to describe nurse champions? (2) What are the disciplinary or theoretical origins of the definitions used to describe nurse champions? (3) What are the defining attributes, antecedents, consequences, references, surrogate terms, and related concepts to nurse champions? (4) What champion training initiatives exist within health care that are attended by nurses? (5) What are the characteristics (eg, target participants and cost) of the nurse champion training initiatives? (6) What competencies are taught in nurse champion training initiatives in health care? (7) What is the evidence that the nurse champion training initiatives in health care increase nurse champions’ knowledge and perceived self-efficacy in performing the nurse champion role? (8) What is the evidence that the nurse champion training initiatives in health care improve nurse champions’ performance and impact? (9) What are the EDI considerations in studies that discuss the nurse champion concept or nurse champion training initiatives? (10) What equity-related factors, as detailed by the PROGRESS-plus (place of residence; race, ethnicity, culture, and language; occupation; gender and sex; religion; education; socioeconomic status; and social capital–plus) framework [50], are used to describe nurse champions and the study’s sample in the included documents? (11) Do any of the studies that evaluate the effectiveness of nurse champion training initiatives integrate EDI considerations? The PROGRESS-plus framework is a widely used framework that outlines categories that potentially influence inequities in health care [50]. There are many research questions, but they pertain to 1 of the 4 research objectives. Specifically, research questions 1 to 3 address objective 1 pertaining to the development of a preliminary conceptual understanding of nurse champions. Similarly, research questions 4-6 address objective 2, which pertains to collating existing nurse champion training initiatives and the competencies that nurse champions gain from their training. Research questions 7 and 8 address objective 3, which pertains to understanding the effectiveness of nurse champion training initiatives. Finally, research questions 9-11 address objective 4 pertaining to examining the EDI considerations related to training and deploying nurse champions. Table 1 details the alignment between the research objectives, research questions, the data extracted, the planned analysis, and how we will publish the results into 4 articles.

**Table 1.** Mapping of research objectives, research questions, data extractions, and analysis plan.

Dissemination	Research objective	Research questions	Data extracted	Analysis plan
Article 1	Develop a preliminary conceptual understanding of nurse champions.	What are the definitions used in the literature to describe nurse champions? What are the disciplinary origins of the definitions used to describe nurse champions? What are the defining attributes, antecedents, consequences, references, surrogate terms, and related concepts to nurse champions?	Definition used to describe nursing champions. Citations and disciplinary origins of definitions used to describe nurse champions. The attributes, antecedents, consequences, references, surrogate terms, and related concepts. Regarding references (context in which the concept has been used), we will extract the (1) health care sectors, (2) type of innovation for, and (3) the recipients of the champions’ facilitation or influence (eg, do champions influence laterally with colleagues or vertically with management). The date of publication of the included studies.	Conventional content analysis [51]. We will code and then combine codes into larger themes to synthesize the attributes, antecedents, consequences, surrogate terms, and related concepts that are pertinent to the concept of nurse champions. We will also develop a definition of the term nurse champion from the extracted attributes of the nurse champion concept. To demonstrate the evolution of the nurse champion, we will analyze the attributes, antecedents, consequences over time, disciplinary origin of definitions, the health care sector, type of innovation, and recipient of champions’ facilitation or influence.
Article 2	Describe the characteristics of existing champion training initiatives in health care that prepare nurse champions and synthesize the competencies that are covered in these champion training initiatives.	What champion training initiatives exist within health care that are attended by nurses? What are the characteristics (eg, target participants, cost) of the nurse champion training initiatives? What competencies are taught in nurse champion training initiatives in health care?	Name of champion training initiative. What sector of health care did the champion training initiative come from? Is the training initiative specific to implementing a particular innovation? What is the innovation? Who are the target participants of the champion training initiative (eg, nurse leaders or clinical nurses)? Cost of training initiative. Location of training initiative. Format of training initiative (eg, in person, virtual, self-paced, and hybrid). Length of training initiative. The main topics covered by the training initiative. Did the authors explicitly outline the competencies (attitudes, judgment, knowledge, and skills) that they intended their participants would gain from their training initiative? The competencies (attitudes, judgment, knowledge, and skills) are emphasized in the champion training initiative. The behaviors that the champion is expected to be able to perform after gaining the competencies from the training initiative.	Summative content analysis [51]. We will group and stratify (using frequency counts and descriptive statistics) the characteristics of the nurse champion training initiatives according to the different health care sectors, the type of innovations, and the target participants of the champion training initiatives. Directed and conventional content analysis [51]: We will organize the data according to AACTT^a^ [52] to demonstrate the attitudes, judgment, knowledge, and skills (competencies) that are linked to the expected behaviors (action) that a champion (actor) enacts to promote the implementation of a particular innovation within a context toward knowledge users (target) and at a specific phase or phases of implementation (time).
Article 3	Synthesize the findings of studies that examined the effectiveness of nurse champion training initiatives in preparing nurses to be effective champions.	What is the evidence that the training initiatives in health care increase nurse champions’ knowledge and perceived self-efficacy in performing the nurse champion role? What is the evidence that the nurse champion training initiatives in health care improve nurse champions’ performance and impact?	Study design of the quantitative studies evaluating the effectiveness of champion training initiatives. Sample size (number of champions attending the training initiative). Measure of knowledge gained by the champion after completing the training initiative. Measure of the champions’ self-efficacy. Measures evaluating the ability of champions to facilitate implementation (eg, number of nurses who interacted with a nurse champion; satisfaction of nurses with the support provided by the champion). Measure of successful implementation (eg, innovation use or impact from the use of the innovation) Reliability and validity of measures. Statistical tests performed. Test statistic or effect size. Statistical significance of results (*P* value).	Meta-analysis if there are more than 2 experimental or quasi-experimental studies that used the same outcome measures with similar populations and interventions [53]. If the outcome measures, population, and interventions are heterogenous, then we will perform a narrative synthesis of the data based on (1) health care sector (eg, acute care, LTC^b^, etc), (2) type of innovation, (3) quantitative research design, (4) outcome measures, (5) significance or lack of significance of results, and (6) quality of studies based on the critical appraisals.
Article 4	Evaluate the extent to which EDI^c^ are considered in studies that define nurse champions and in studies describing or evaluating nurse champion training initiatives attended by nurse champions.	What are the EDI considerations in studies that discuss the nurse champion concept or nurse champion training initiatives? What equity-related factors as detailed by the PROGRESS-plus^d^ framework (1) are used to describe nurse champions and the study’s sample in the included documents? Do any of the studies that evaluate the effectiveness of nurse champion training initiatives integrate EDI considerations?	Definition of nurse champions in the context of the implementation of innovations in equity-deserving populations. The context in which champions facilitate the implementation of innovations for equity-deserving populations. The name and description of the champion training initiatives that include EDI considerations. EDI considerations are present in champion training initiatives. The equity-related factors used to describe champions and the sample. The positionality of the authors of the documents.	Conventional and summative content analysis [54]. We will perform conventional content analysis to synthesize themes in the extracted data pertaining to EDI considerations in the included documents that discussed the nurse champion concept or nurse champion training initiatives and nurse champion training initiatives’ effectiveness. We will conduct summative content analysis to describe (1) the demographics of the nurse champions and the study sample according to the PROGRESS-plus framework [50]; and (2) the number of included documents that detailed EDI considerations.

a AACTT: action, actor, context, target, time.

b LTC: long-term care.

c EDI: equity, diversity, and inclusion.

d PROGRESS-plus: place of residence; race, ethnicity, culture, and language; occupation; gender and sex; religion; education; socioeconomic status; and social capital–plus.

### Identifying Relevant Studies

#### Overview

We performed preliminary searches on Google and Google Scholar to identify papers that are relevant to our study. We developed a search strategy on MEDLINE in consultation with a health science librarian (Victoria Cole) and informed by the key terms we identified from the preliminary online searches. The search strategy includes both Boolean phrases and Medical Subject Headings (MeSH) terms. The key terms in the search strategy include (1) champions and (2) nursing and synonyms (Table 2 for the preliminary search strategy). A second health science librarian evaluated the search strategy using the peer review of electronic search strategy (PRESS) checklist [55] (Multimedia Appendix 1). WJS translated and applied the search strategy to the other databases.

We searched the published literature through 8 health care, education, and management databases: Business Source Complete, CINAHL, Embase, ERIC, MEDLINE, Nursing and Allied Health Premium, PsycINFO, and Scopus. We searched education and management databases to capture articles about nurse champions in nursing education or management. Regarding gray literature, we searched for theses and dissertations from ProQuest and Theses Canada. Other gray literature sources that we will search include (1) documents from the Canadian Agency For Drugs And Technologies In Health Grey Matters, (2) documents from champion training initiative or program websites such as the Registered Nurses’ Association of Ontario’s Best Practice Champions Network [56] and the Strengthening a Palliative Approach in Long-Term Care Champions’ page [57], and (3) government and policy documents through Google search of the keywords champion* and nurs*. We will also contact authors of included documents on an as-needed basis for any other documents detailing champion competencies or the characteristics of their champion training initiatives when more information is required. We will assess the reference lists of included documents, and we will search the list of articles that have cited the included documents using Scopus and Web of Science. We will also evaluate systematic reviews (identified through the online database searches) on champions for any other pertinent articles.

**Table 2.** Preliminary search strategy on MEDLINE.

Number	Query	Search results from April 24, 2025, n
1	champion*.tw,kf.	11,896
2	nurs*.tw,kf.	580,298
3	exp Practice Patterns, Nurses'/ or exp Nurse’s Role/ or exp Licensed Practical Nurses/ or exp Nurses/ or exp nursing, practical/ or exp faculty, nursing/ or exp nursing staff/	210,885
4	2 or 3	654,444
5	1 and 4	1326

#### Inclusion Criteria

We will include documents that meet these criteria (1) primary studies (quantitative, qualitative, and mixed-methods) and gray literature; (2) documents that describe individuals who are explicitly referred to as nurse champions; (3) documents written in any language; and (4) documents published in 1970 and onwards as champions were first identified by Schon’s study on radical military innovations [14,36] in the 1960s and 1970s, and this cut off was previously used in recent reviews on champions [7,9]; (5) documents that provide a definition or describe the concept nurse champion, describe a nurse champion training initiative and the competencies emphasized by the nurse champion training initiative, evaluate a nurse champion training initiative, or discuss EDI considerations pertaining to deployment or training of nurse champions; and (6) documents that are related to implementation of innovations or evidence-based practices.

#### Exclusion Criteria

We will exclude documents that (1) are not available in full text; (2) are explicitly only about other knowledge translation roles; (3) are review papers but relevant review papers about champions will be tagged to have their references screened; (4) are conference abstracts but relevant conference abstracts will be tagged and evaluated for relevant articles; (5) are about veterinary nurse champions; (6) are not set in a health care context; and (7) are clinical trial registrations or protocol registration. Any clinical trial registration or protocol registrations that might be relevant to answering the research questions will be tagged, and we will appraise these registrations for any relevant documents.

### Study Selection

WJS uploaded the downloaded records from the databases to Covidence, and this system automatically deduplicated the records. Another researcher (Letitia Nadalin-Penno, Megan Greenough, or Priscilla Packiam) and WJS will independently read and screen the titles and abstracts and then full texts in accordance with the inclusion and exclusion criteria. The 2 reviewers (Letitia Nadalin-Penno, Megan Greenough, or Priscilla Packiam and WJS) will pilot the inclusion and exclusion criteria on 100 titles and abstracts; screening will begin once there is ≥75% agreement (κ higher than 0.75) between the reviewers, which determines substantial agreement between the 2 reviewers (Letitia Nadalin-Penno, Megan Greenough, or Priscilla Packiam and WJS) [58]. The 2 reviewers (Letitia Nadalin-Penno, Megan Greenough, or Priscilla Packiam and WJS) will resolve conflicts through discussion or through consultation of a third senior research member (IDG or JES). We will translate titles and abstracts and full-text documents not written in English using the University of Ottawa’s enterprise instance of Microsoft Copilot [54]. We will use our research networks to identify individuals who can confirm the accuracy of the translation of the included non-English documents before we move on to data extraction and quality appraisal for these documents. If we cannot identify a native speaker in our networks, we will hire a professional translator to translate the article into English once we confirm that it meets inclusion.

To address research questions 1-3 related to research objective 1 (to increase conceptual understanding of the nurse champion concept), we will include documents that provide a definition of nurse champions or detail the attributes, antecedents, consequences, related terms, and reference of use of the nurse champion concept and we will place a tag named “concept analysis” for these documents on Covidence [59] during screening. To address research questions 4-6 related to research objective 2 (to collate nurse champion training initiatives and the nurse champion competencies emphasized within the training initiatives), we will include documents that detail training initiatives that are intended to prepare nurse champions. We will place a tag named “competencies” for these documents on Covidence [59]. To address research questions 7 and 8 related to research objective 3 (to synthesize the findings of studies on the effectiveness of the nurse champion training initiatives in increasing nurse champions’ self-efficacy or improving their impact), we will prioritize inclusion of randomized controlled trials and quasi-experimental studies that evaluate the effectiveness of champion training initiatives in training nurse champions. However, we will also include other primary nonexperimental quantitative studies that evaluate the correlation between attending a nurse champion training initiative and the nurse champions’ knowledge and perceived self-efficacy in performing the nurse champion’s role, and the nurse champions’ performance and impact. Although these nonexperimental studies do not determine the effectiveness [60], they can highlight existing relationships. We will also include documents that evaluate the effectiveness of training of nurse champions and other knowledge translation roles (eg, physician champions, knowledge broker, or opinion leaders) if details specific to nurse champions can be extracted separately. We will place a tag named “effectiveness” for the documents pertaining to the effectiveness of champion training initiatives. To address research questions 9-11 pertaining to research objective 4 (to evaluate the extent that EDI considerations are present in studies about the nurse champion concept or nurse champion training initiatives), we will place a tag named “EDI” for the documents that detail EDI considerations in relation to either the nurse champion concept or the champion training initiatives.

### Data Charting

We will develop a data charting form on Covidence. The data charting form will include (1) study characteristics: year of publication, study’s authors, discipline or field of the primary author, study design according to authors’ and extractor’s interpretation of design, and health care context; (2) characteristics of nurse champions (eg, sex, gender, ethnicity, etc); (3) the innovation being implemented; (4) the intended recipients or users of the innovation; (5) nurse champions’ behaviors; (6) definitions used to describe nurse champions and the disciplinary or theoretical origins of the definitions; (7) attributes, antecedents, consequences, references, surrogate, and related concepts to the nurse champion concept; (8) characteristics of champion training initiatives: target participants, cost, length, accessibility (eg, how often are they offered; online or in person), and content of champion training initiatives (eg, main topics covered); (9) competencies emphasized by the champion training initiatives and their definitions; (10) how studies evaluated champions’ effectiveness (eg, study design and sample size); (11) number of champions, outcome measures, reliability and validity of measures, statistical tests, test statistic, effect size, and significance of results; (12) EDI considerations in included studies pertaining to the nurse champion concept or the nurse champion training initiatives; and (13) equity-related factors used to describe champions and the study sample in accordance with the PROGRESS-plus framework [50]. Table 1 describes the data planned to be extracted in relation to the research objectives and questions. Data charting will be completed independently and in duplicate by 2 reviewers (Letitia Nadalin-Penno, Megan Greenough, and Priscilla Packiam or WJS). The 2 reviewers (Letitia Nadalin-Penno, Megan Greenough, and Priscilla Packiam or WJS) will pilot the data charting form on 2‐3 papers. The data charting form may be updated as the study is conducted due to the iterative nature of data charting according to Peters et al [47]. The reviewers will resolve conflicts in data charting through consensus or through consultation with a third senior research member (IDG or JES).

### Critical Appraisal

Two research members (Letitia Nadalin-Penno, Megan Greenough, or Priscilla Packiam and WJS) will perform an independent critical appraisal of the included studies. Even though it is not a necessary step in scoping reviews, we will evaluate and report the quality of documents included in the scoping review, as we will be extracting and synthesizing the results reported in these documents. The following JBI critical appraisal tools will be used (1) analytical cross-sectional, (2) case control, (3) case reports, (4) cohort study [61], (5) expert opinion [62], (6) quasi-experimental studies [60], (7) qualitative research [63], (8) narrative [62], (9) policy [62], and (10) randomized controlled trials critical appraisal tools [60] and the Mixed Methods Appraisal Tool for mixed methods studies [64]. The quality of the evidence will be described qualitatively; for instance, the authors will highlight strengths and limitations of the included articles as per the JBI critical appraisal tools and the Mixed Methods Appraisal Tool.

### Stage 5: Collating, Summarizing, and Reporting Results

We will report on the (1) study characteristics (year of publication, study’s authors, the discipline or field of the primary author, the study design, and the health care context), (2) the characteristics of nurse champions (eg, sex, gender, ethnicity), (3) the innovation being implemented, and (4) the characteristics of the intended recipients or users of the innovation (profession, sex, gender, etc).

To achieve our 4 research objectives, we sought to conduct 4 subanalyses. The first subanalysis addresses the first research objective and research questions 1-3 pertaining to the development of a preliminary conceptual understanding of nurse champions. We will follow Rodgers’ evolutionary concept analysis approach [39] to guide this subanalysis. We will inductively analyze data using conventional content analysis [51] to determine the following regarding nurse champions: (1) attributes of the nurse champion concept (defining characteristics of the concept that makes it identifiable across different contexts and allows for identification of situations in which the concept may be applicable [39]), (2) antecedents (necessary circumstances or characteristics that precede the concept [39]), (3) consequences (circumstances or characteristics that are produced following the existence of the concept), (4) surrogate terms (other words or phrases used to describe the concept other than the ones that are used or results from the analysis [39]), and (5) related terms (associated but distinct words or phrases relating to nurse champions [39]). Conventional content analysis follows an inductive approach: (1) data are coded, (2) codes are then clustered to form subcategories, and (3) these subcategories are then aggregated to create broader categories. The clustering of data into codes and subcategories is based on a subjective interpretation of similarities and differences in the text data [51]. WJS will code the data independently, and another researcher (Letitia Nadalin-Penno, Megan Greenough, and Priscilla Packiam) will check the coding. The coding will also be discussed with senior research members (AV, IDG, JES, or GH) during weekly to biweekly meetings. We will develop a definition of the term nurse champion according to the extracted attributes of the nurse champion concept [39]. We will use simple frequency counts to outline how many documents support a certain code or category. To demonstrate the evolution of the concept nurse champion, we will perform summative content analysis [39] by counting the number of times that each attribute, antecedent, and consequence of the nurse champion concept were mentioned in included documents according to the (1) date of publication, (2) disciplinary origin of definitions or the primary author’s disciplines, (3) the theoretical origins (eg, conceptual frameworks, theoretical frameworks, or theories) of the definitions, (4) the health care sector, (5) type of innovation, and (6) the recipient of nurse champions’ facilitation or influence.

The second subgroup analysis addresses the second research objective and research questions 4-6 pertaining to describing existing champion training initiatives in health care that prepare nurse champions and the competencies that are emphasized in these training initiatives. We will use summative content analysis [51] to report the characteristics of the nurse champion training initiatives according to different health care sectors, the type of innovations, and the target participants of the champion training initiatives using frequency counts or descriptive statistics. We will use conventional content analysis [51] to code and categorize the champion competencies emphasized in the training initiatives as attitudes, judgment, knowledge, or skills. We will construct definitions for each competency based on the data extracted. A second individual will check the data coding. We will use directed content analysis [51] to organize the data pertaining to nurse champions’ behaviors according to action, actor, context, target, time [52], to demonstrate the attitudes, judgment, knowledge, or skills (competencies) that are linked to the expected behaviors (action) that a nurse champion (actor) enacts to promote the implementation of a particular innovation within a context toward knowledge users (target) and at a specific phase or phases of implementation (time). The research team will have weekly to biweekly meetings to discuss the extracted competencies and constructed definitions.

The third subgroup analysis addresses the third research objective and research questions 7 and 8 pertaining to the effectiveness of the champion training initiatives in increasing the champions’ knowledge, self-efficacy, performance, and impact toward implementation. We will conduct a meta-analysis if there are more than 2 experimental or quasi-experimental studies that used the same outcome measures with similar populations and interventions [53,60]. The outcomes of interest, in accordance with Shea’s conceptual model, intending to guide research on the activities and effects of innovation champions [7], will be (1) change in champions’ knowledge and perceived self-efficacy in performing the champion role, and (2) improvement in the champions’ performance and impact on the implementation process and outcome. If the outcome measures, populations, or interventions are heterogenous, then we will perform a narrative synthesis [60] of the data based on (1) health care sector (eg, acute care), (2) type of innovation, (3) quantitative research design, (4) outcome measures, (5) significance or lack of significance of results, and (6) quality of studies based on the critical appraisals. The meta-analysis or narrative synthesis will be performed by WJS; senior research members (AV, IDG, JES, or GH) will evaluate the analysis during weekly to biweekly meetings.

To address the fourth research objective and research questions 9-11, we will use conventional content analysis to synthesize the themes pertaining to the EDI considerations reported in the documents that discussed the nurse champion concept or nurse champion training initiatives and nurse champion training initiatives’ effectiveness. We will also conduct summative content analysis [51] to describe the demographics of the nurse champions in accordance with equity-related factors detailed by the PROGRESS-plus framework [50] using frequency counts and descriptive statistics. The PROGRESS-plus framework is a widely used framework that describes socially stratifying categories that influence inequities in health care [50]. We will also use summative content analysis to describe the number of included documents that discussed EDI considerations pertaining to the nurse champion concept or nurse champion training initiatives (using frequency counts or descriptive statistics). WJS will perform the initial coding that will be verified by another research team member (Letitia Nadalin-Penno, Megan Greenough, and Priscilla Packiam). The analysis will also be evaluated by senior research team members (AV, IDG, JES, or GH) during weekly to biweekly meetings.

### Review Quality

We will use the PRISMA-ScR (Preferred Reporting Items for Systematic reviews and Meta-Analyses extension for Scoping Reviews) checklist [65] and the Evidence-Based Checklist for Improving Scoping Review Quality [66] to ensure review quality and transparent reporting. We will also use the Standards for Reporting Qualitative Research checklist [67] for reporting the subanalyses pertaining to the nurse champion concept and the champion training initiatives, as these subanalyses involve qualitative analyses of textual data. We will use the PRISMA (Preferred Reporting Items for Systematic reviews and Meta-Analyses) checklist [68] to ensure transparent reporting of the subanalysis pertaining to the effectiveness of the champion training initiatives. We will use the Synthesis Without Meta-analysis in systematic reviews reporting checklist [69] if we conduct narrative synthesis, rather than a meta-analysis of the data extracted from studies reporting the effectiveness of champion training initiatives. We will report these checklists as an additional file in the resulting articles. A preliminary PRISMA-ScR checklist can be found in Checklist 1.

## Results

**Figure 1.**
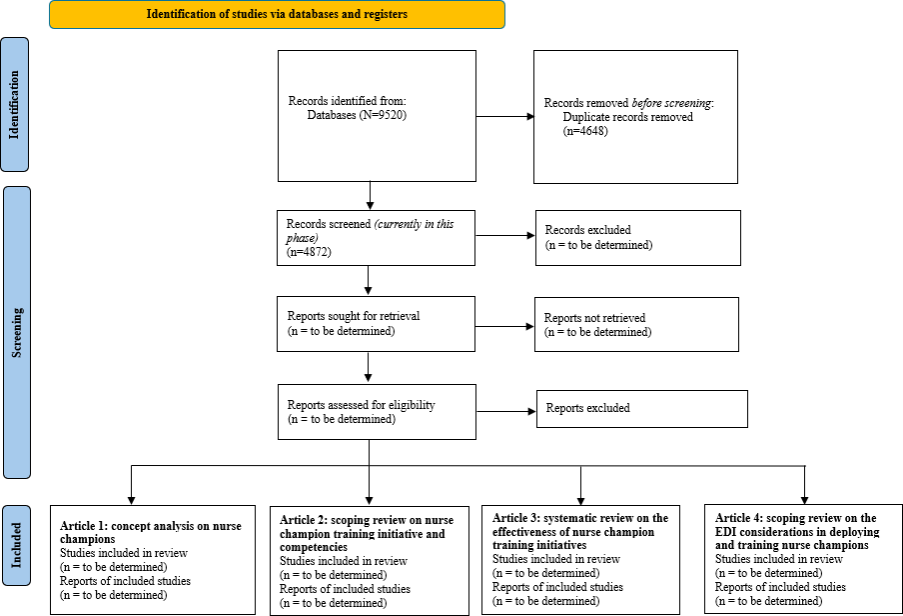
PRISMA (Preferred Reporting Items for Systematic reviews and Meta-Analyses) flowchart. EDI: equity, diversity, and inclusion.

We expect to present the results of the scoping review in 2026. As of January 2026, we have applied our search strategy on the databases we have selected and detailed above, piloted our inclusion and exclusion criteria for title and abstract and full-text screening, and partially completed title and abstract screening. Currently, our online database search has identified 4872 titles and abstracts (Figure 1). We will present the results of this scoping review across 4 peer-reviewed articles, with each article addressing one of our 4 research objectives and the research questions related to each of the objectives. As depicted in Figure 1 (an in-progress PRISMA diagram), we will specify the total number of records screened, the total number included in the scoping review, and the total number of studies included for the subsequent articles derived from the same unified dataset. We will cross-reference the articles included in the 4 articles to ensure accurate reporting of the total number included in the scoping review.

## Discussion

Many authors have reported the importance of deploying nurse champions to enable implementation of best practices in health care [6,8,26-30]. The current literature pertaining to the concept of health care champions has advanced knowledge pertaining to the following: (1) differentiated health care champions from other concepts that enable implementation based on their theoretical origin and the mechanisms in which they exert social influence [15,36], (2) described the plethora of personal attributes, skills, and behaviors that health care champions are reported to perform [6,15,37], and (3) illustrated conceptual models that described the relationships between different constructs that influence how health care champions enable change [7,38]. Since the use and application of concepts can change over time and with different contexts [39,40], there is an opportunity to explore how the nurse champion concept is defined across different health care contexts. Furthermore, there is a lack of knowledge pertaining to the training of nurse champions, the competencies that nurse champions should possess, and whether these training initiatives are effective [8,12]. There is also a lack of understanding of EDI considerations in research about nurse champions and health care champions [38]. This scoping review will (1) advance conceptual understanding of nurse champions, (2) collate existing nurse champion training initiatives and synthesize the competencies emphasized in these training initiatives, (3) synthesize the evidence pertaining to the effectiveness of the training initiatives that prepare nurse champions, and (4) summarize the EDI considerations pertaining to the nurse champion concept or the EDI considerations embedded in nurse champion training initiatives. This research will provide guidance on how to improve existing nurse champion training initiatives, which may result in better-prepared nurse champions and implementation success. Although the focus of this research is limited to nurse champions, it is possible that some of the findings may be transferable to other disciplines in health care.

## Supplementary material

10.2196/80204 Multimedia Appendix 1 PRESS - nurse champions scoping review.

10.2196/80204 Checklist 1 PRISMA-ScR checklist.

## Abbreviations

EDI: equity, diversity, and inclusion
JBI: Joanna Briggs Institute
PRESS: Peer Review of Electronic Search Strategy
PRISMA: Preferred Reporting Items for Systematic reviews and Meta-Analyses
PRISMA-ScR: Preferred Reporting Items for Systematic reviews and Meta-Analyses extension for Scoping Reviews
PROGRESS-plus: place of residence; race, ethnicity, culture, and language; occupation; gender and sex; religion; education; socioeconomic status; and social capital–plus

## References

[1] Braithwaite J, Glasziou P, Westbrook J (2020). The three numbers you need to know about healthcare: the 60-30-10 challenge. BMC Med.

[2] Squires JE, Cho-Young D, Aloisio LD (2022). Inappropriate use of clinical practices in Canada: a systematic review. CMAJ.

[3] Graham ID, Logan J, Harrison MB (2006). Lost in knowledge translation: time for a map?. J Contin Educ Health Prof.

[4] Harrison MB, Graham ID (2021). Knowledge Translation in Nursing and Healthcare: A Roadmap to Evidence-Informed Practice.

[5] Harvey G, Loftus-Hills A, Rycroft-Malone J (2002). Getting evidence into practice: the role and function of facilitation. J Adv Nurs.

[6] Miech EJ, Rattray NA, Flanagan ME, Damschroder L, Schmid AA, Damush TM (2018). Inside help: an integrative review of champions in healthcare-related implementation. SAGE Open Med.

[7] Shea CM (2021). A conceptual model to guide research on the activities and effects of innovation champions. Implement Res Pract.

[8] Santos WJ, Graham ID, Lalonde M, Demery Varin M, Squires JE (2022). The effectiveness of champions in implementing innovations in health care: a systematic review. Implement Sci Commun.

[9] Astorino Nicola J, Nataliansyah MM, Lopez-Olivo MA (2024). Champions to enhance implementation of clinical and community-based interventions in cancer: a scoping review. Implement Sci Commun.

[10] Wood K, Giannopoulos V, Louie E (2020). The role of clinical champions in facilitating the use of evidence-based practice in drug and alcohol and mental health settings: a systematic review. Implement Res Pract.

[11] Hall AM, Flodgren GM, Richmond HL (2021). Champions for improved adherence to guidelines in long-term care homes: a systematic review. Implement Sci Commun.

[12] Jolliffe L, Lannin NA, Larcombe S, Major B, Hoffmann T, Lynch E (2025). Training and education provided to local change champions within implementation trials: a rapid systematic review. Implement Sci.

[13] Chakrabarti AK (1974). The role of champion in product innovation. Calif Manage Rev.

[14] Schon DA (1963). Champions for radical new inventions. Harv Bus Rev.

[15] Cranley LA, Cummings GG, Profetto-McGrath J, Toth F, Estabrooks CA (2017). Facilitation roles and characteristics associated with research use by healthcare professionals: a scoping review. BMJ Open.

[16] Kuehl H, Mabry L, Elliot DL, Kuehl KS, Favorite KC (2013). Factors in adoption of a fire department wellness program: champ-and-chief model. J Occup Environ Med.

[17] Shaw EK, Howard J, West DR (2012). The role of the champion in primary care change efforts: from the State Networks of Colorado Ambulatory Practices and Partners (SNOCAP). J Am Board Fam Med.

[18] Soo S, Berta W, Baker GR (2009). Role of champions in the implementation of patient safety practice change. Healthc Q.

[19] Jennings G (2019). Introducing learning disability champions in an acute hospital. Nurs Times.

[20] Lee SJC, Higashi RT, Inrig SJ (2017). County-level outcomes of a rural breast cancer screening outreach strategy: a decentralized hub-and-spoke model (BSPAN2). Transl Behav Med.

[21] Luz S, Shadmi E, Admi H, Peterfreund I, Drach-Zahavy A (2019). Characteristics and behaviours of formal versus informal nurse champions and their relationship to innovation success. J Adv Nurs.

[22] Maier MA, Brem A (2018). What innovation managers really do: a multiple-case investigation into the informal role profiles of innovation managers. Rev Manag Sci.

[23] Titler MG, Everett LQ (2001). Translating research into practice. Considerations for critical care investigators. Crit Care Nurs Clin North Am.

[24] Melnyk BM, Fineout-Overholt E, Stillwell SB, Williamson KM (2010). Evidence-based practice: step by step: the seven steps of evidence-based practice. Am J Nurs.

[25] Aloisio LD, Graham N, Grinspun D (2023). Indicators to measure implementation and sustainability of nursing best practice guidelines: a mixed methods analysis. Heliyon.

[26] Chisholm A, Russolillo A, Carter M (2025). Advancing evidence-based practice through the knowledge translation challenge: nurses’ important roles in research, implementation science and practice change. J Adv Nurs.

[27] Campbell J (2008). The effect of nurse champions on compliance with keystone intensive care unit sepsis-screening protocol. Crit Care Nurs Q.

[28] Anand KJS, Eriksson M, Boyle EM (2017). Assessment of continuous pain in newborns admitted to NICUs in 18 European countries. Acta Paediatr.

[29] Bentz CJ, Bayley KB, Bonin KE (2007). Provider feedback to improve 5A’s tobacco cessation in primary care: a cluster randomized clinical trial. Nicotine Tob Res.

[30] Engel M, van Zuylen L, van der Ark A, van der Heide A (2021). Palliative care nurse champions’ views on their role and impact: a qualitative interview study among hospital and home care nurses. BMC Palliat Care.

[31] White CL (2011). Nurse champions: a key role in bridging the gap between research and practice. J Emerg Nurs.

[32] Siebeck OS, Hoving C (2024). Characteristics of a successful nurse peer champion in the implementation of innovative digital technologies in hospitals: a qualitative study. PEC Innov.

[33] Sperling D, Shadmi E, Drach-Zahavy A, Luz S (2022). Nurse champions as street-level bureaucrats: factors which facilitate innovation, policy making, and reconstruction. Front Psychol.

[34] Ploeg J, Skelly J, Rowan M (2010). The role of nursing best practice champions in diffusing practice guidelines: a mixed methods study. Worldviews Evid Based Nurs.

[35] Harvey G, Kitson A (2016). PARIHS revisited: from heuristic to integrated framework for the successful implementation of knowledge into practice. Implement Sci.

[36] Thompson GN, Estabrooks CA, Degner LF (2006). Clarifying the concepts in knowledge transfer: a literature review. J Adv Nurs.

[37] Bonawitz K, Wetmore M, Heisler M (2020). Champions in context: which attributes matter for change efforts in healthcare?. Implement Sci.

[38] Morena AL, Gaias LM, Larkin C (2022). Understanding the role of clinical champions and their impact on clinician behavior change: the need for causal pathway mechanisms. Front Health Serv.

[39] Rodgers BL, Knafl KA (2000). Concept Development in Nursing: Foundations, Techniques, and Applications.

[40] Rodgers BL (1989). Concepts, analysis and the development of nursing knowledge: the evolutionary cycle. J Adv Nurs.

[41] Straus S, Tetroe J, Bhattacharyya O, Zwarenstein M, Graham I (2013). Knowledge Translation in Health Care: Moving from Evidence to Practice.

[42] Mrayyan MT, Abunab HY, Abu Khait A (2023). Competency in nursing practice: a concept analysis. BMJ Open.

[43] McKeganey SN (2000). The rise and rise of peer education approaches. Drugs Educ Prev Policy.

[44] Heidenreich S, Breukers S (2020). Who is telling whose story? The effectiveness of peer-to-peer approaches as inclusive participatory interventions towards sustainability. Sustain Prod Consum.

[45] Hendy J, Barlow J (2012). The role of the organizational champion in achieving health system change. Soc Sci Med.

[46] Koenig AM, Eagly AH, Mitchell AA, Ristikari T (2011). Are leader stereotypes masculine? A meta-analysis of three research paradigms. Psychol Bull.

[47] Peters MD, Godfrey C, McInerney P, Munn Z, Tricco AC, Khalil H, Aromataris E, Lockwood C, Porritt K, Pilla B, Jordan Z (2020). JBI Manual for Evidence Synthesis.

[48] Arksey H, O’Malley L (2005). Scoping studies: towards a methodological framework. Int J Soc Res Methodol.

[49] The JBI approach in evidence implementation. JBI.

[50] O’Neill J, Tabish H, Welch V (2014). Applying an equity lens to interventions: using PROGRESS ensures consideration of socially stratifying factors to illuminate inequities in health. J Clin Epidemiol.

[51] Hsieh HF, Shannon SE (2005). Three approaches to qualitative content analysis. Qual Health Res.

[52] Presseau J, McCleary N, Lorencatto F, Patey AM, Grimshaw JM, Francis JJ (2019). Action, actor, context, target, time (AACTT): a framework for specifying behaviour. Implement Sci.

[53] Deeks JJ, Higgins JP, Altman DG, Higgins JPT, Thomas J, Chandler J, Cumpston M, Li T, Page MJ, Welch VA, Group CSM (2019). Cochrane Handbook for Systematic Reviews of Interventions.

[54] Using Microsoft Copilot at uOttawa. University of Ottawa.

[55] McGowan J, Sampson M, Salzwedel DM, Cogo E, Foerster V, Lefebvre C (2016). PRESS peer review of electronic search strategies: 2015 guideline statement. J Clin Epidemiol.

[56] Best Practice Champions Network. Registered Nurses’ Association of Ontario.

[57] Champions. SPA LTC.

[58] What is inter-rater reliability?. Covidence.

[59] (2025). Better systematic review management. Covidence.

[60] Tufanaru C, Munn Z, Aromataris E, Campbell J, Hopp L, Aromataris E, Munn Z (2020). JBI Reviewer’s Manual.

[61] Moola S, Munn Z, Tufanaru C, Aromataris E, Sears K, Sfetcu R, Aromataris E, Munn Z (2020). JBI Manual for Evidence Synthesis JBI.

[62] McArthur A, Klugarova J, Yan H, Florescu S (2020). JBI Manual for Evidence Synthesis JBI.

[63] Lockwood C, Munn Z, Porritt K (2015). Qualitative research synthesis: methodological guidance for systematic reviewers utilizing meta-aggregation. Int J Evid Based Healthc.

[64] Hong QN, Gonzalez-Reyes A, Pluye P (2018). Improving the usefulness of a tool for appraising the quality of qualitative, quantitative and mixed methods studies, the mixed methods appraisal tool (MMAT). J Eval Clin Pract.

[65] Tricco AC, Lillie E, Zarin W (2018). PRISMA extension for scoping reviews (PRISMA-ScR): checklist and explanation. Ann Intern Med.

[66] Cooper S, Cant R, Kelly M (2021). An evidence-based checklist for improving scoping review quality. Clin Nurs Res.

[67] O’Brien BC, Harris IB, Beckman TJ, Reed DA, Cook DA (2014). Standards for reporting qualitative research: a synthesis of recommendations. Acad Med.

[68] Page MJ, McKenzie JE, Bossuyt PM (2021). The PRISMA 2020 statement: an updated guideline for reporting systematic reviews. BMJ.

[69] Campbell M, McKenzie JE, Sowden A (2020). Synthesis without meta-analysis (SWiM) in systematic reviews: reporting guideline. BMJ.

