# Supplementary material for: Understanding the Nurse Champion Concept and the Training Initiatives for Nurse Champions: Protocol for a Scoping Review
Source: JMIR Res Protoc. 2026 Jan 14;15:e80204. doi: 10.2196/80204 (PMC12808872; doi:10.2196/80204)
Supplement: Multimedia Appendix 1 [file resprot-v15-e80204-s001.docx]

# Additional File 1 – PRESS Checklist

***PRESS Guideline* — Search Submission & Peer Review Assessment**

**SEARCH SUBMISSION: THIS SECTION TO BE FILLED IN BY THE SEARCHER**

| Searcher: Wilmer S | Email: |  |
| --- | --- | --- |
| Date submitted: Jul 14 | Date requested by: Jul 25 |  |

**Systematic Review Title:**

**Understanding the Nurse Champion Concept and the Training Initiatives for Nurse Champions: A Scoping Review Protocol**

This search strategy is …

| X | My PRIMARY (core) database strategy — First time submitting a strategy for search question and database |
| --- | --- |
|  | My PRIMARY (core) strategy — Follow-up review NOT the first time submitting a strategy for search question and database. If this is a response to peer review, itemize the changes made to the review suggestions |
|  | SECONDARY search strategy— First time submitting a strategy for search question and database |
|  | SECONDARY search strategy — NOT the first time submitting a strategy for search question and database. If  this is a response to peer review, itemize the changes made to the review suggestions |

**Database**

(i.e., MEDLINE,CINAHL…): *[mandatory]*

**MEDLINE**

**Interface**

(i.e., Ovid, EBSCO…): *[mandatory]*

**OVID**

**Research Question**

(Describe the purpose of the search) *[mandatory]*

1. To develop a preliminary conceptual understanding of nurse champions.
2. To describe the characteristics of existing champion training initiatives in healthcare that prepare nurse champions and synthesize the competencies that are covered in these champion training initiatives.
3. To synthesize the findings of studies that examined the effectiveness of nurse champion training initiatives in preparing nurses to be effective champions.
4. To evaluate the extent to which equity, diversity, and inclusion (EDI) are considered in studies that define nurse champions and in studies describing or evaluating nurse champion training initiatives attended by nurse champions.

**PICO Format**

(Outline the PICOs for your question — i.e., Patient, Intervention, Comparison, Outcome, and Study Design — as applicable)

| **P** | Nurse champions |
| --- | --- |
| **I** | initiatives, training, preparation |
| **C** | n/a |
| **O** | Effectiveness, competence, nurse champions’ knowledge and perceived self-efficacy, performance, impact, integration of EDI considerations |
| **S** | No limits on design,languages, years |
|  |  |

**Inclusion Criteria**

(List criteria such as age groups, study designs, etc., to be included) *[optional]*

**Exclusion Criteria**

(List criteria such as study designs, date limits, etc., to be excluded) *[optional]*

**Was a search filter applied?**

Yes No
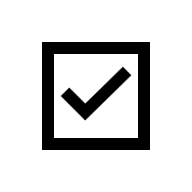


**If YES, which one(s) (e.g., Cochrane RCT filter, PubMed Clinical Queries filter)? Provide the source if this is a published filter.** *[mandatory if YES to previous question* — *textbox]*

n/a

Other notes or comments you feel would be useful for the peer reviewer**?**

**This search focuses specifically on nurse champions, not enablers, implementation leaders, change agents, coaches, opinion leaders, knowledge broker, facilitators, and linking agents**

Please copy and paste your search strategy here, exactly as run, including the number of hits per line. ***[mandatory]***

**Database: Ovid MEDLINE**

**# Query Results from 24 Apr 2025**

**1 champion*.tw,kf. 11,896**

**2 nurs*.tw,kf. 580,298**

**3 exp Practice Patterns, Nurses’/ or exp Nurse’s 210,885**

**Role/ or exp Licensed Practical Nurses/ or exp**

**Nurses/ or exp nursing, practical/ or exp**

**faculty, nursing/ or exp nursing staff/**

**4 2 or 3 654,444**

**5 1 and 4 1,326**

**PEER REVIEW ASSESSMENT: THIS SECTION TO BE FILLED IN BY THE REVIEWER**

|  | Reviewer: Tamara Rader | Email: tamara.rader@gmail.com | Date completed: Jul 25 | | |
| --- | --- | --- | --- | --- | --- |
|  |  |  |  | | |
|  | **1. TRANSLATION** |  |  | | |
| A -­‐No revisions | | X |  |  |  |
| B -­‐ Revision(s) suggested | | ☐ |  |  |  |
| C -­‐ Revision(s) required | | ☐ |  |  |  |

The authors have described their rationale for focusing on nurse champions, even though the concept has been described generally and also in other health disciplines. This review will focus on the concept of a nurse champion and build on work on similar concepts described in other disciplines.

The approach relies on the most broad terms to capture more specific, descriptive terms:

- For example <https://pubmed.ncbi.nlm.nih.gov/35777942/> includes the MeSH term
  Nurses, Community Health* - which would be captured by the most broad term Exp Nurses/

**2. BOOLEAN AND PROXIMITY OPERATORS**

| A -­‐No revisions | X |
| --- | --- |
| B -­‐ Revision(s) suggested | ☐ |
| C -­‐ Revision(s) required | ☐ |

If “B” or “C,” please provide an explanation or example:

**3. SUBJECT HEADINGS**

| A -­‐No revisions | ☐ |
| --- | --- |
| B -­‐ Revision(s) suggested | X |
| C -­‐ Revision(s) required | ☐ |

I would add the MeSH term Nursing/ to the term in Line 3 to be consistent in the approach to be as broad as possible.

**4. TEXT WORD SEARCHING**

| A -­‐No revisions | X |
| --- | --- |
| B -­‐ Revision(s)suggested | ☐ |
| C -­‐ Revision(s) required | ☐ |

**5. SPELLING, SYNTAX, AND LINE NUMBERS**

| A -­‐No revisions | X |
| --- | --- |
| B -­‐ Revision(s)suggested | ☐ |
| C -­‐ Revision(s) required | ☐ |

If “B” or “C,” please provide an explanation or example:

**6. LIMITS AND FILTERS**

| A -­‐No revisions | X |
| --- | --- |
| B -­‐ Revision(s) suggested | ☐ |
| C -­‐ Revision(s) required | ☐ |

If “B” or “C,” please provide an explanation or example:

No filters used.

OVERALL EVALUATION (Note: If one or more “revision required” is noted above, the response below must be “revisions required”.)

| A -­‐No revisions | ☐ |
| --- | --- |
| B -­‐ Revision(s) suggested | X |
| C -­‐ Revision(s) required | ☐ |

Additional comments:
